# Deep Learning-Based Design Method for Acoustic Metasurface Dual-Feature Fusion

**DOI:** 10.3390/ma17092166

**Published:** 2024-05-06

**Authors:** Qiang Lv, Huanlong Zhao, Zhen Huang, Guoqiang Hao, Wei Chen

**Affiliations:** School of Electrical and Electronic Engineering, Wuhan Polytechnic University, Wuhan 430048, China; huanlzhao@outlook.com (Q.L.); zhenhuang@whpu.edu.cn (Z.H.); guoqhao@163.com (G.H.); 18627216595@163.com (W.C.)

**Keywords:** metasurface, deep neural network, acoustic field modulation, inverse design, genetic algorithm

## Abstract

Existing research in metasurface design was based on trial-and-error high-intensity iterations and requires deep acoustic expertise from the researcher, which severely hampered the development of the metasurface field. Using deep learning enabled the fast and accurate design of hypersurfaces. Based on this, in this paper, an integrated learning approach was first utilized to construct a model of the forward mapping relationship between the hypersurface physical structure parameters and the acoustic field, which was intended to be used for data enhancement. Then a dual-feature fusion model (DFCNN) based on a convolutional neural network was proposed, in which the first feature was the high-dimensional nonlinear features extracted using a data-driven approach, and the second feature was the physical feature information of the acoustic field mined using the model. A convolutional neural network was used for feature fusion. A genetic algorithm was used for network parameter optimization. Finally, generalization ability verification was performed to prove the validity of the network model. The results showed that 90% of the integrated learning models had an error of less than 3 dB between the real and predicted sound field data, and 93% of the DFCNN models could achieve an error of less than 5 dB in the local sound field intensity.

## 1. Introduction

Acoustic metasurfaces are artificially designed materials with superior acoustic manipulation capabilities at subwavelength scales [1,2]. It was used in underwater communications, sound absorption and noise reduction, acoustic stealth, and other fields [3,4,5,6,7]. Researchers had now used a variety of methods to design metasurfaces. For example, designing specific structures such as labyrinths and spatial curl structures [8,9,10,11,12,13,14], establishing a theoretical model that could be numerically calculated [15,16], a combination of different materials such as water and silicone rubber [17,18,19], and building codable and reprogrammable metasurfaces [20,21,22]. All of the above hypersurface design methods had been studied based on finite element simulations and high intensity iterations, and this repetitive process consumed a lot of the researchers’ time.

Traditional metasurface design methods were long, complex, time-consuming, and labor-intensive and required deep acoustic knowledge from the designer. Deep learning could construct end-to-end mappings from metasurfaces to sound field distributions, saving time and resources while reducing the designer’s requirements for acoustic knowledge. Some researchers had introduced deep learning algorithms into the design process of acoustic metasurfaces, which in turn enabled the inverse design between structural parameters and acoustic fields [23,24,25]. For example, Zhao et al. used a convolutional neural network model to establish a mapping of the local acoustic field to the metasurface phase gradient and finally realized the regional control of the local acoustic field [26]. Gao et al. used the transfer matrix to establish the acoustic sink model and then used CNN to realize the effective prediction of the sound absorption curve [27]. Li et al. proposed a tandem neural network method to reverse-engineer the phase of metasurface elements, which achieved an energy loss of more than 10 dB in the echo direction of the sound wave [24]. Sajedian et al. relied on a dual deep Q learning network (DDQN) to find the right material type and the best ensemble design [28]. Machine learning was also being used to encode metasurfaces, which manipulated the sound field by arranging logic units into specific sequences [29,30]. The above data-driven deep learning-based approach has contributed significantly to the work on the reverse design of metasurfaces. A purely data-driven approach requires high data quality and lacks common sense, so predictions may not make physical sense [31]. In the process of making model predictions for deep learning, the results of model predictions based solely on data-driven models may appear to be beyond the physical range. Finding a reasonable condition to constrain the predictive ability of the model and, thus, constructing a one-to-one mapping between the hypersurface parameters and the acoustic field distribution is a challenge. How to obtain a physical feature and use it to implement constraints on the predictive power of a network model was a difficult issue.

The aforementioned method focused more on the metasurface design but did not focus on the precise tuning of the local sound field. To achieve precise control of the local sound field. In this paper, due to the lack of input information, the learning function of a single model was insufficient, and it was difficult to establish the mapping relationship between the structural parameters of the sound field and the distribution of the sound field intensity. Therefore, an ensemble learning method was constructed to establish the forward mapping relationship between physical properties, structural parameters, and acoustic field data. It could generate datasets from built models. A dual-feature-driven model based on a convolutional neural network (DFCNN) was proposed. When the physical characteristics of the acoustic field were combined with the complex nonlinear characteristics between the acoustic field and the structural parameters for metasurface reverse design, it could simultaneously combine nonlinear features and extracted physical features. By dividing the sound field distribution into channels, this preprocessing of the input data was combined with network modeling in such a way that the approach could be implemented for channels with smaller or larger ranges. Furthermore, the genetic algorithm was used to optimize the parameters to avoid the loss of performance caused by the mismatch of parameter settings. The DFCNN proposed in this paper was to further mine the features present in the data itself, not a complex analysis of theoretical or physical models, which simplified the acoustic expertise requirements and tedious repetitive experiments to a certain extent. It provided a feasible solution for acoustic field modulation using acoustic metasurfaces.

## 2. Physical Modeling of the Localized Acoustic Field on the Metasurfaces

The theoretical basis for acoustic metasurfaces was the generalized Snell’s law [32], it could be expressed as:

(1) $$\sin\theta_{r}-\sin\theta_{i}=\frac{1}{k_{0}}\frac{d\Phi \left(x\right)}{dx}$$

where *θ_r_* was the angle of reflection, *θ_i_* was the angle of incidence, *k*_0_ was the wave velocity, and *d*Φ(*x*)/*dx* was the phase gradient along the tangential direction of the interface.

In order to achieve effective control of sound waves and reduced sound energy loss, all incident sound waves should be projected onto the metasurface, so the perpendicular incidence (*θ_i_* = 0) should be fully projected and fully reflected on the upper and lower surfaces of the metasurface. When a sound wave was incident perpendicular to the metasurface at the speed of sound *c*_0_, its ideal density distribution *ρ*(*x*) satisfied Equation (2) [33]:

(2) $$\rho \left(x\right)=\left(\frac{1}{2h}\sin\left(\theta_{r}\right)x+C_{0}\right)\rho_{0},\;0\leq x\leq L$$

where *L* was the length of the metasurface, *C*_0_ was the integration constant, *θ_r_* was the reflection angle, *ρ*_0_ was the density of the incident medium, *h* was the normal thickness of the metasurface, and *x* was the position. The density distribution *ρ*(*x*) of a material was the decisive parameter affecting its reflected acoustic field. The artificial periodic structure could not achieve the ideal continuous change of physical property parameters. In order to approximate the continuous material density distribution of the theoretical metasurface, the theoretical metasurface could be discretized into *m* elements (*i* = 1, 2,..., *n*) along the length direction. The density of each discrete element was characterized by the density *ρ_i_* at its central position [34,35].

**Figure 1:**
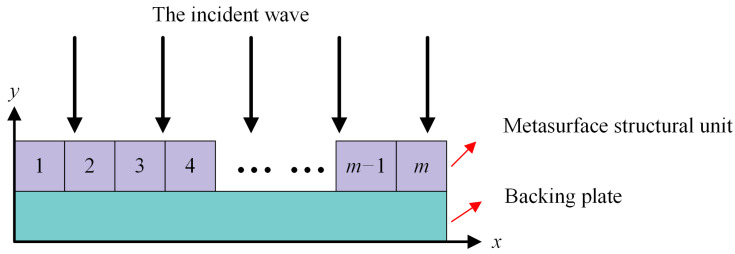
Schematic diagram of the sound field model.

In this paper, starting from the idea of parametric modeling, there was no structural restriction on the metasurface structural elements, and the simplified parameters were used instead of the metasurface structural elements to establish the physical model of the acoustic field, as shown in Figure 1. Firstly, the plane wave perpendicular incident underwater was used as the background field, and then the metasurface was composed of *m* metasurface structural elements arranged in the positive direction of *x*, and the metasurface was covered on the surface of the backing plate. When the plane wave is incident on the metasurface in the opposite direction of *y*, the reflected waves generated by *m* metasurface structural units interact with each other to form the entire reflected sound field. The physical structural parameters of each structural unit could be different. In this paper, the physical structural parameters of each unit would be obtained according to the gradient arrangement to satisfy the intensity characteristics of the desired sound field distribution. The effective parameter set **P** was the gradient value *g* and the density *ρ* of the first structural unit.

## 3. Method

### 3.1. Forward Model Design Based on Ensemble Learning

#### 3.1.1. Dataset Preparation

**Figure 2:**
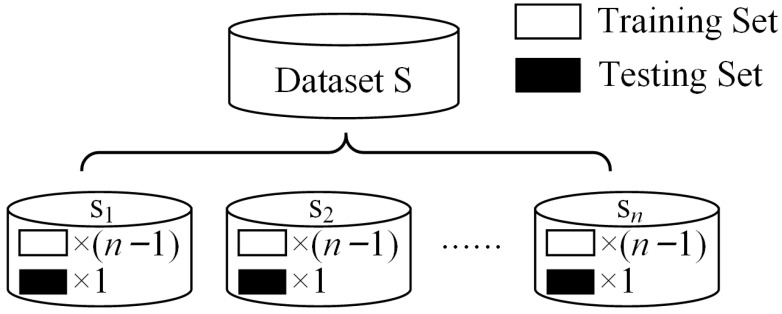
N-fold cross validation method.

In the forward model, a homogeneous ensemble learning model was composed of *n* base models. The dataset was composed of the metasurface physical structure parameter **P** and the corresponding target acoustic field intensity matrix **I**. The structural unit density took values in the range [1/3, 3] (kg/m^3^). The gradient value *g* needed to satisfy that the structural unit density could not exceed the range of values. In performing the division of the dataset, the n-fold cross-validation method was used according to the number of base models. The dataset was divided into *n* groups, where the *n* − 1 group was the training set and the remaining 1 group was used as the test set, as shown in Figure 2. The n-fold cross-validation method allowed each base model to use a different sample set. More data features could be learned.

#### 3.1.2. Ensemble Learning Models

The forward design could construct a mapping relationship between the metasurface structure and the target sound field strength. The ensemble learning model constructed a one-to-one mapping of the nonlinear relationship between the metasurface structure and the target sound field intensity. Firstly, the sample set **S** was divided into *n* different subsample sets according to the n-fold cross-validation method. Then the FCN was trained, and the FCN must be maintained until the FCN converges during training. The trained model was used as a candidate for the base model. Under the same parameter training conditions, the fast convergence and small loss function were selected as the base model. Homogeneous models could generate completely different model weights by setting different network parameters. Differences in model weights meant learning different features. The metamodel was trained. Through the error analysis of the predicted sound field and the target sound field, it was tested whether the forward design between the parameters and the sound field was realized.

**Figure 3:**
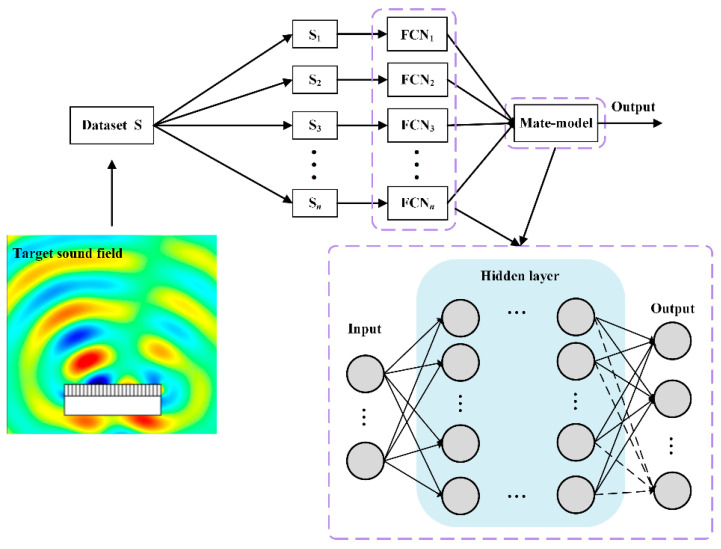
A framework for ensemble learning.

The model framework of ensemble learning was shown in Figure 3, and the ensemble learning model consisted of *n* first-level base models and one second-level element model. The target sound field strength was used as a primary base model input. The output of each base model was the input to the metamodel. The output of the metamodel was the final output of the entire ensemble learning model. The sample set of each first-level base model was divided into a training set and a testing set after the total sample set was scrambled, which ensured that each base model could learn as many local region features as possible in the sample space. The metamodel could reassign weights to the features learned by each base model so that the erroneous features learned by the base model can be corrected to a certain extent.

#### 3.1.3. Algorithm Details and Discussion

The entire deep neural network model was built using the PyTorch deep learning framework, and all the Python code of the network model was run using Spyder 5.1.5 on the Anaconda3_5.2.0 platform. The ensemble learning model was built with *n* = 5 base models. The 5-fold cross-validation method was used to divide the data set. Table 1 shows the specific network parameters of each base model.

After the integrated learning framework was constructed, it could be seen from Figure 3 that the mapping relationship **I** between the hypersurface physical structure parameter **P** and the sound field distribution matrix could be expressed as Equation (3):

(3) $$I=F^{M}\left(\sum_{i}^{N}f_{i}^{S}\left(P\right)\right),\left(i=1,2,\ldots ,5\right)$$

where **I** was the acoustic field distribution matrix, *F^M^*(·) denoted the metamodel, $f_{i}^{S}$(·) represented the *i* base model, and **P** represented the hypersurface physical structure parameters. The model training was measured using the mean square error loss function (MSE) as in Equation (4):

(4) $$L_{MSE}=\frac{1}{N}\sum_{i=1}^{N}{\left(y_{i}-{\hat{y}}_{i}\right)}^{2}$$

where *y_i_* was the true value and *ŷ* represented the predicted value. Due to the integration of multiple models, combined with Equation (3), the loss function could be further rewritten as:

(5) $$L_{EL}=\sum_{i=1}^{n}\omega_{i}\left(\frac{1}{N}\sum_{j=1}^{N}{\left(I_{j}-{\hat{I}}_{j}\right)}^{2}\right)$$

where *n* was the total number of base models, *ω_i_* was the weight of the *i* base model, the specific weight value could be assigned by the meta-model, *N* was the number of sample sets, *I_j_* was the true sound field value, and ${\hat{I}}_{j}$ was the predicted sound field value.

**Figure 4:**
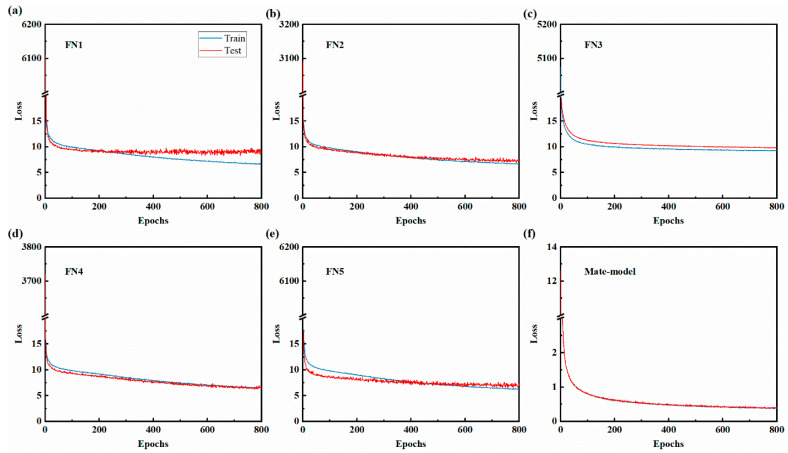
Change in loss curve. (**a**–**e**) represent the base model loss function curve, (**f**) represent the ensemble learning loss function curve.

The variation of the loss function with batch for the test and training sets of each model in the integrated learning framework is shown in Figure 4, which shows that the loss functions of both the training and test sets of the base model are decreasing and finally reach convergence near 10. The loss function of the training and test sets of the whole integrated learning model finally converges around 0.5. The loss function value decreased by about 9.5 compared to the single model loss function value, and the degree of oscillation was relatively smooth. The difference between the loss function values of the test set and the training set is about 0.02, showing high consistency, which indicates that the model has strong generalization ability.

### 3.2. Reverse Design Method Based on DFCNN

#### 3.2.1. Data Preprocessing

The entire reflected acoustic field was affected by the coupling relationship between all metasurface structural elements, so the local acoustic field could not be considered an isolated part. Local sound field manipulation is needed to take into account the influence of the entire sound field [36]. It was difficult to directly use data-driven deep learning methods to realize the inverse mapping relationship between structural parameters and sound field intensity. In this paper, the characteristics of sound field data were further mined.

In the entire sound field, the coupling relationship between the various metasurface structural elements was reflected in the sound field. The whole sound field could be divided into multi-channel equidimensional sound field matrix **V**, and the connection between each sound field matrix **V** could make up for the lack of physical characteristics of direct data drive to a certain extent. Considering that it was not possible to divide the entire sound field into equidimensional channels, the first and last segments of the entire sound field were filled with 0. 0, which has no meaning; it did not bring noise or information interference. The sound field after channel division could be expressed as:

(6) $$Ι=\sum_{i=1}^{N}V_{i},(i=1,2,\ldots ,N)$$

where **I** represented the entire sound field, **V***_i_* was the sound field matrix channels, and *N* was the number of channels divided. Therefore, the reconstructed dataset was composed of the metasurface structural element parameter **P** and the acoustic field matrix **I**. In the dataset, 10,000 sets of data were derived from the real sound field data, and the remaining 20,000 sets of data were obtained by data augmentation by the aforementioned ensemble learning method, but the noise data will inevitably be mixed after the data enhancement.

#### 3.2.2. DFCNN Network

The dataset was built to compensate for the lack of physical characteristics in a direct data-driven approach. The feature information in DFCNN came from two parts: One was the nonlinear mapping relationship learned by direct data drive, and the other part was the physical feature information that makes up for it. The structure parameter **P** could be expressed as follows:

(7) $$P=F\left(I\right)⊗H\left(\sum_{i=1}^{N}V_{i}\right)$$

where *F*(·) and *H*(·) represented the nonlinear mapping relationship and the physical feature information relationship, respectively. There was a correlation between the multi-channel sound field matrices, and they were not equally important for the participation of the local sound field. This correlation was a linear or nonlinear weighting relationship. The two features could be expressed as follows:

(8) $$P=\Psi \left(F\left(I\right)⊗H\left(\sum_{i=1}^{N}\beta_{i}V_{i}\right)\right)$$

where *β_i_* was the weight relation, Ψ(·) was a feature fusion relationship.

**Figure 5:**
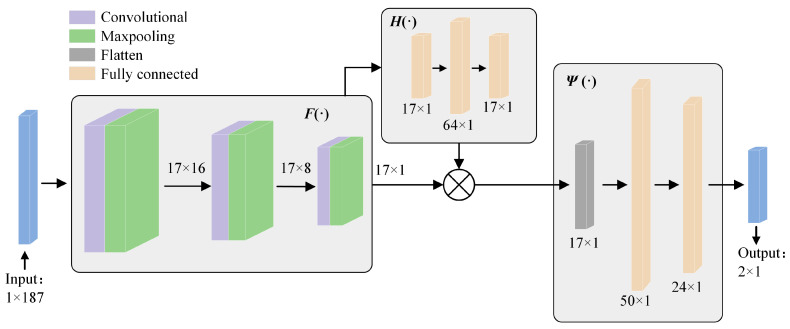
DFCNN framework.

Based on this, the network model architecture is shown in Figure 5. The model consisted of three parts, *F*(·) and *H*(·), representing the nonlinear mapping relationship and the physical feature information relationship extraction network, respectively, Ψ(·) for the feature fusion network. Finally, the prediction parameters were obtained.

*H*(·) The purpose of this study was to obtain physical feature information and extract the weight relationship *β* of multiple channels. This part was built with the multi-channel attention mechanism borrowed [37]. During model training, the complex coupling relationship made it difficult for the weight relationship ***β*** to focus on the desired local sound field channel. The workaround was to additionally add a parameter matrix ***λ***, which could heuristically guide the model to focus on the desired local acoustic field channels. This parameter matrix ***λ*** obeyed the standard normal distribution N(0, 1), which only needed to satisfy the desired local acoustic field at which the channel is slightly larger than the other channels.

#### 3.2.3. Algorithm Details

The choice of model parameters often directly affected the prediction performance. The loss function value could evaluate the parameter setting good or bad, and this method required a certain amount of experience in parameter tuning to optimize the model performance. For example, the learning rate could be dynamically adjusted by the Adam optimization algorithm, but an inappropriate initial learning rate directly led to a decrease in the iteration speed of the model and the consumption of computational resources. To address the above situation, this paper used a genetic algorithm to optimize the model parameters. The key parameters in the network model were taken as genes. The genes of population individuals underwent selection, crossover, and mutation operations. The optimal part of the population in each generation was retained, after continuous optimization iterations until the best optimized parameters were reached.

**Figure 6:**
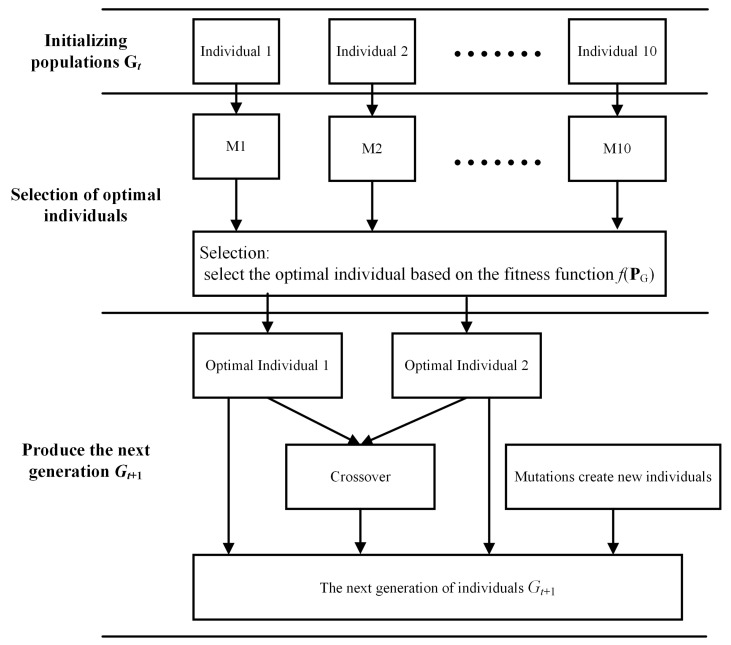
Genetic algorithm process.

Figure 6 shows the operation process of the GA algorithm. The GA algorithm first establishes the parameter set **P***_G_* that needs to be optimized by the genetic algorithm and establishes the fitness function *f*(**P**_G_). Input individual parameters into the network model M. Excluding the selected set of parameters, each network model used the same parameters, including datasets, partition scales, and so on. The dataset size used was 10,000 sets, and the partition ratio was 8:2. After that, the population G*_t_* is initialized, and the value **P*_G_****_t_* of the initial parameter set is determined at the same time. Then, the fitness of the population individuals is calculated, and the optimal individuals are retained. In this paper, two optimal individuals are retained. The existing optimal individuals are selected. The crossover operation produces an individual. Simultaneous execution of the mutation operation produces 7 individuals. Then the next generation of population G*_t+_*_1_ is generated, and the optimal individual solution is output when the set algebra is reached. DFCNN is trained based on the optimal set of parameters obtained by the GA algorithm.

When building GA, P*_G_* included lr (learning rate), activation function, and optimizer. The fitness function *f*(P*_G_*) was used to judge the merits of each generation, and the formula was as follows:

(9) $$f\left(P_{G}\right)=\frac{1}{N}\sum_{i=1}^{N}\left(\left(y_{i}-{\hat{y}}_{i}\right)\leq \varepsilon \right)$$

where *N* was the number of sample sets, and *ε* was the set threshold, which was set to 0.05 in this paper. The GA parameter optimization problem could be described as:

(10) $$\begin{array}{l} \mathrm{Find}:{\;P}_{G}\\ \mathrm{Objective}:\;\max\;f\left(P_{G}\right)\\ \mathrm{Subject}\;\mathrm{to}:\;\mathrm{lr}\in \left[10^{-3},10^{-1}\right],\\ \mathrm{Activation}\;\mathrm{Function}\in \left[\mathrm{ReLU},\mathrm{LeakyReLU},\mathrm{PReLU}\right],\\ \mathrm{Optimizer}\in \left[\mathrm{Adam},\mathrm{SGD},\mathrm{Nadam}\right] \end{array}$$

In Equation (10), the range of values for lr was set. In the selection of activation functions, the three activation functions had the advantages of improving gradient vanishing and increasing computational speed. Both Leaky ReLU and PReLU complemented the deficiencies of ReLU. In the selection of optimizers, three commonly used optimizers were also selected. Nadam was an improved version of Adam.

In this paper, the local sound field channel was set to 85°~95°. V had a dimension of 1 × 11. By adding 3 zeros at the beginning and end of the original dataset, the completed dataset could be divided into 17 channels, as shown in Figure 5. The input is a dimension of 1 × 187, and the *F*(·) network consists of convolutional and pooling layers. The first layer of the convolutional network has a convolutional kernel size of 11 with a step size of 11, the second layer of the convolutional network has a convolutional kernel size of 5 with a step size of 1, the third layer of the convolutional network has a convolutional kernel size of 5 with a step size of 1, and the pooling layers are all chosen to have maximal pooling with sizes of 2, 5, and 4, respectively, with the specific parameters as shown in Figure 5. *H*(·) network consists of 3 layers of fully connected layers, and the Ψ(·) network consists of 3 layers of fully connected layers, and finally the model output is obtained. Fully connected layers, and Ψ(·) network consists of flatten layer and 2 fully connected layers to get the final model output. The model used the mean square error loss function (MSE) as the evaluation index, and its formula was as follows Equation (4). Table 2 shows the parameters of the DFCNN model and GA.

**Figure 7:**
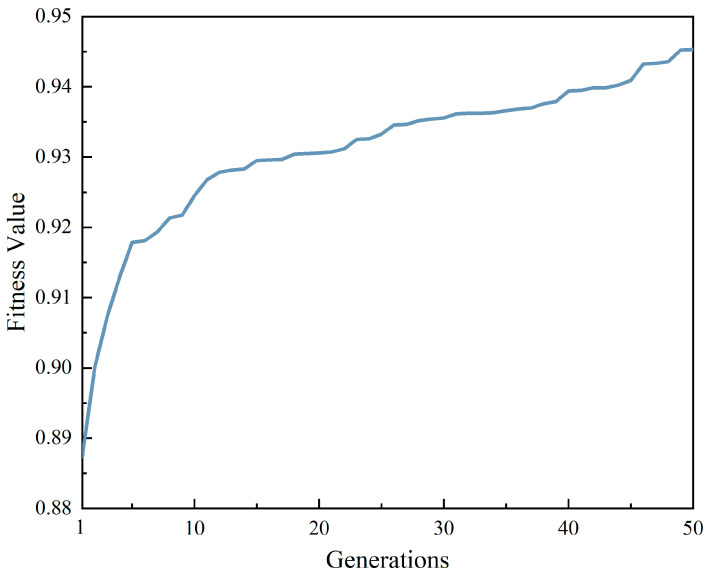
Fitness value curve.

**Figure 8:**
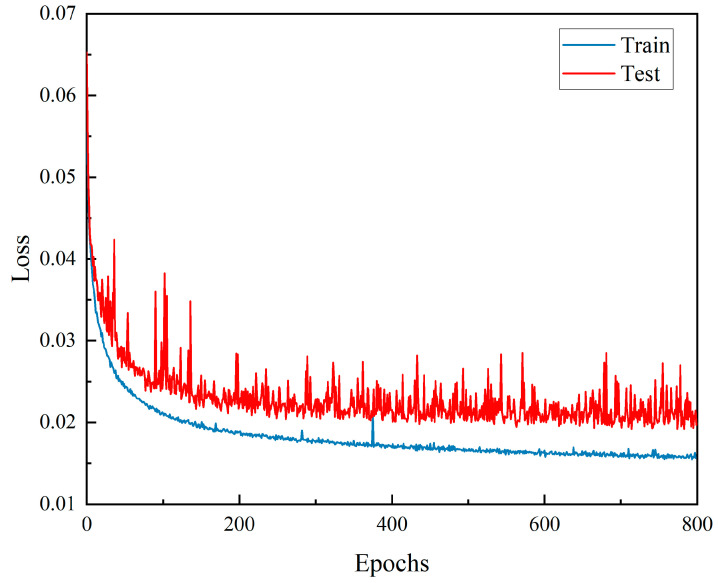
DFCNN loss function curve.

The parameters of the genetic algorithm model were set according to the parameters in Table 2, and the fitness function value curve after 50 generations of population replacement was shown in Figure 7, and it could be seen from the figure that the population fitness function value was also increasing with the increase of population reproduction generations. The maximum value of the fitness function was 0.945, and the corresponding parameters were 0.00471, Adam, and PReLU, respectively. In order to further improve the generalization ability of the model, the DFCNN model was set and retrained according to the parameters obtained by GA optimization. The loss function was still the mean square error loss function (MSE). As shown in Figure 8, after 800 epochs of the DFCNN model, the loss function curve gradually converges, with the loss function of the training set and the loss function of the test set convergent to about 0.015. The difference between the training set and the test set after the convergence of the loss function is about 0.005, which means that the fitting ability of the model has reached a good level.

## 4. Discussion of Results

### 4.1. Ensemble Learning Model Results

**Figure 9:**
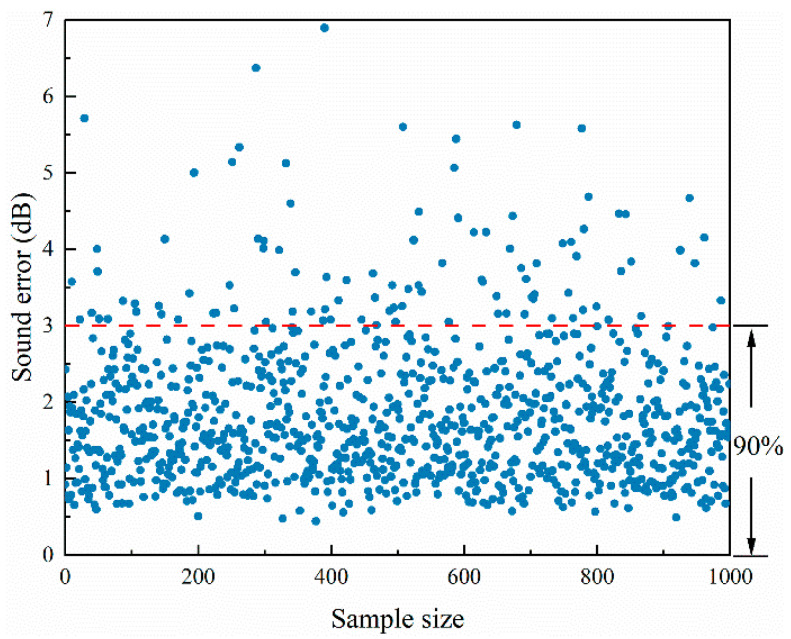
Sound field intensity error plot.

In order to better verify the generalization ability of the model, an additional 1000 sets of data were used for model testing. In Figure 9, the vertical coordinate is the average error value over the whole sound field, and the horizontal coordinate is the number of samples, with 90% of the samples having an average error of less than 3 dB over the whole sound field.

**Figure 10:**
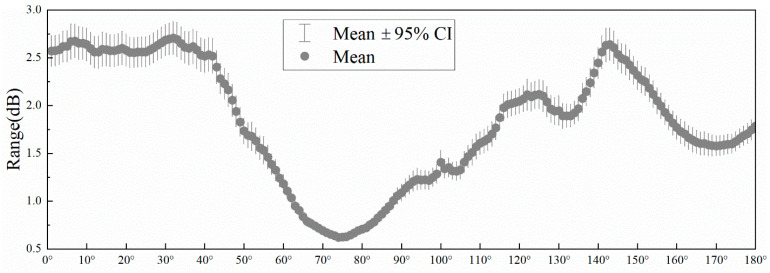
Error distribution interval for a single angle of the sound field.

In Figure 10, the horizontal coordinate is the sound field angle, and the vertical coordinate is the range of the mean 95% confidence interval. The range of the mean 95% confidence interval for the entire sound field angle is less than 3 dB, and more than 50% of the sound field angles can be realized with an error of less than 2 dB. In Figure 10, the echo direction is around 90°, compared with other parts of the mean confidence interval, which is located in the range of 1.5~2 dB reduction. This obvious reduction shows that the model can learn the structural parameters of the sound field data from the complex coupling relationship exhibited, which also provides strong support for the feasibility of the subsequent mining of the physical characteristics of the acoustic field information.

**Figure 11:**
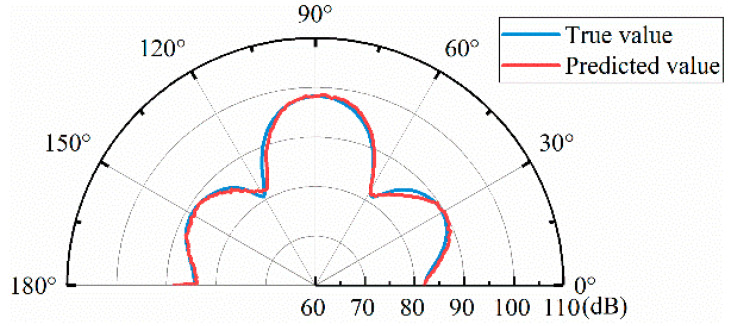
Examples of true and predicted sound field results.

A random set of 1000 sets of data tested by the model is shown in Figure 11 for the comparison between the real and predicted sound fields. It can be seen that the predicted sound field is in good agreement with the real sound field, which can be used for data enhancement. As can be seen from the figure, the forward model based on ensemble learning can predict the sound field distribution corresponding to the physical structure parameters of the metasurface, but the prediction error near the reflection direction is small, about 0.75 dB, and the prediction error in other directions is large, about 2 dB.

### 4.2. DFCNN Model Results

**Figure 12:**
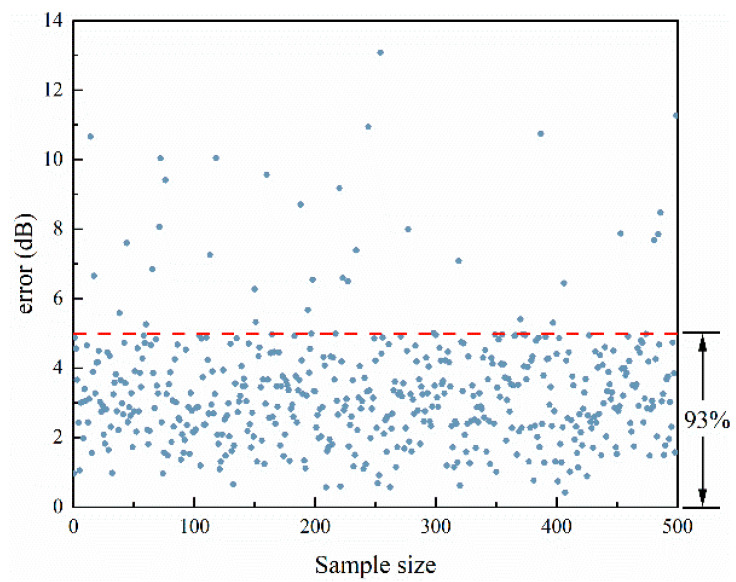
Error plot of local sound field intensity.

**Figure 13:**
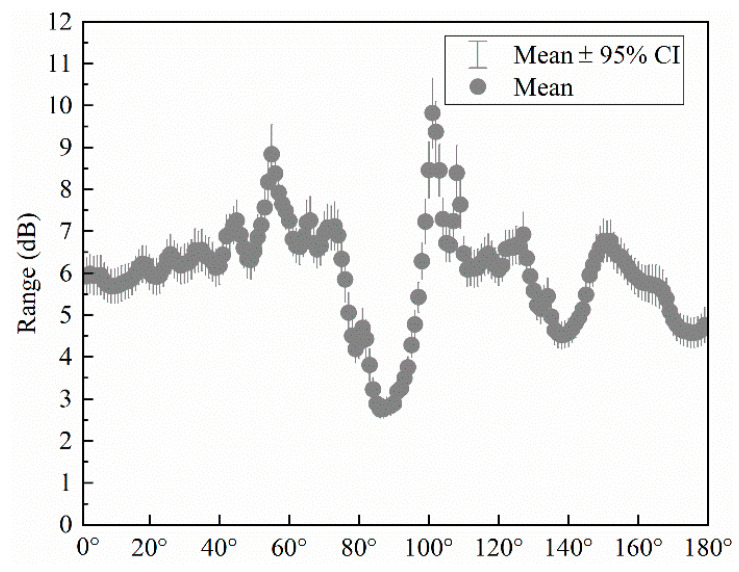
Prediction of the sound field error distribution interval plot.

In order to prove the effectiveness of the DFCNN model, an additional 500 sets of data that did not participate in the training were used for further verification. Of the 500 validated sets of data, 93% of the average error of local acoustic field strength was less than 5 dB, as shown in Figure 12. Analyzing the error distribution of the entire sound field, as shown in Figure 13, it can be seen that the error in the local sound field is much smaller than the rest of the sound field error, and the 95% confidence interval range of the mean sound field error is less than 3 dB in the adjusted local interval. Most of the sound field regions outside the tuning interval have an error of about 4 dB to 7 dB, and some sound field regions have an error of about 9 dB, which shows that the DFCNN has a better tuning effect on the local sound field.

**Figure 14:**
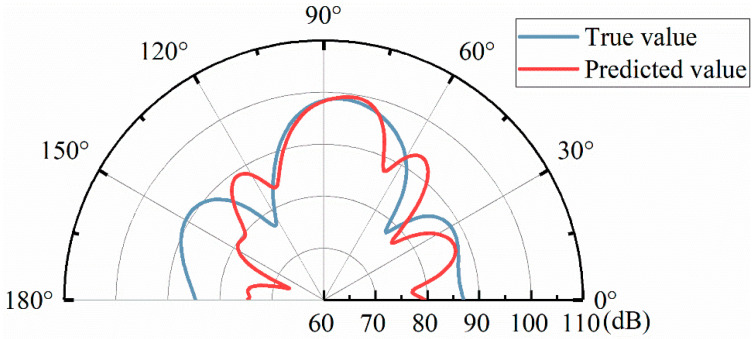
The results of the real and predicted sound fields under the DFCNN model.

A group of 500 sets of data tested by the model is randomly selected, and the comparison between the real sound field and the predicted sound field is shown in Figure 14, which shows that the prediction of the local sound field has good results. Through the above analysis, it can be found that the use of a variety of feature information in the process of model training can have a good fitting effect on the complex coupling relationship between the whole sound field and the local sound field, in which the addition of physical feature information has a constraining effect on the local sound field, has a certain anti-interference ability to the noise mixed in the data enhancement process in the data set, and has good robustness.

In this paper, 93% of the data error can be achieved within 3 dB for the control of local sound field intensity, which proves that we can realize the control of sound field intensity and can be applied to the stealth, detection, and underwater communication of underwater vehicles.

## 5. Conclusions

In this paper, the ensemble learning method was used to construct a forward mapping model between the parameters of the physical structure of the metasurface and the acoustic field, which could obtain a large amount of acoustic field data according to the parameters. A convolutional neural network-based dual-feature fusion model (DFCNN) was proposed, which used the model to mine the physical feature information of the sound field while maintaining the original high-dimensional nonlinear features. It then realized the dual-feature fusion driven by data features and physical features, and the parameters were optimized with the help of a genetic algorithm. The results showed that in ensemble learning, 90% of the true and predicted sound field data could be less than 3 dB error, and the 95% confidence interval of the mean angle error of a single true and predicted sound field was within 3 dB. In the DFCNN model, 93% of the local sound field strength could be achieved with a data error of less than 5 dB. It provided a new way for local sound field control. However, the model constructed in this paper divides the forward and reverse design into two processes, which can be optimized into one step in future work, and the method proposed in this paper is still insufficient for the control of global sound field intensity, which is also the place that needs to be modified in the follow-up work.

## Figures and Tables

**Table 1:** Base model network parameter settings.

Submodel	lr	Batch_Size	Epoch	Dropout	Optimizer	Number of Layers and Neurons Setting
FCN1	0.005	512	800	0.1	Adam	2→724→1000→650→400→200→181
FCN2	0.005	256	800	0.1	Adam	2→724→1000→650→400→200→181
FCN3	0.005	512	800	0.1	SGD	2→724→1000→600→400→300→181
FCN4	0.01	512	800	0.1	Adam	2→700→1000→650→400→200→181
FCN5	0.005	256	800	0.2	Adam	2→724→1000→650→400→200→181

**Table 2:** Parameter settings.

Name	Specific Settings
Dataset	30,000
Criteria for division	80%, 20%
batch_size	256
epoch_GA	150
Number of iterations	50
Population size	10
mutation_rate	0.4
lr	10 (−3, −1)
Activation function	ReLU, Leaky ReLU, PReLU
Optimizer	Adam, SGD, Nadam
epoch_DFCNN	800

## Data Availability

The raw data supporting the conclusions of this article will be made available by the authors on request.
